# Antibacterial effects of chitosan-based hydrogels containing *Trachyspermum ammi* essential oil on pathogens isolated from dogs with otitis externa

**DOI:** 10.1186/s12917-024-03971-7

**Published:** 2024-04-01

**Authors:** Niloofar Jelokhani Niaraki, Shahram Jamshidi, Bahar Nayeri Fasaei, Seyed Mehdi Joghataei

**Affiliations:** 1 Department of Microbiology and Immunology, Faculty of Veterinary Medicine, University of Tehran, Tehran, Iran; 2 Department of Internal Medicine, Faculty of Veterinary Medicine, University of Tehran, Tehran, Iran

**Keywords:** Essential oil, *Trachyspermum ammi*, Otitis externa, Nanoparticles, Chitosan, Hydrogels

## Abstract

**Background:**

Growing antibiotic resistance has made treating otitis externa (OE) increasingly challenging. On the other hand, local antimicrobial treatments, especially those that combine essential oils (EOs) with nanoparticles, tend to be preferred over systemic ones. It was investigated whether Ajwain (*Trachyspermum ammi*) EO, combined with chitosan nanoparticles modified by cholesterol, could inhibit the growth of bacterial pathogens isolated from OE cases in dogs. In total, 57 dogs with clinical signs of OE were examined and bacteriologically tested. Hydrogels of Chitosan were synthesized by self-assembly and investigated. EO was extracted (Clevenger machine), and its ingredients were checked (GC-MS analysis) and encapsulated in chitosan-cholesterol nanoparticles. Disc-diffusion and broth Micro-dilution (MIC and MBC) examined its antimicrobial and therapeutic properties.

**Results:**

*Staphylococcus pseudintermedius* (49.3%) was the most common bacteria isolated from OE cases, followed by *Pseudomonas aeruginosa* (14.7%), *Escherichia coli* (13.3%), *Streptococcus canis* (9.3%), *Corynebacterium auriscanis* (6.7%), *Klebsiella pneumoniae* (2.7%), *Proteus mirabilis* (2.7%), and *Bacillus cereus* (1.3%). The investigation into the antimicrobial properties of Ajwain EO encapsulated in chitosan nanoparticles revealed that it exhibited a more pronounced antimicrobial effect against the pathogens responsible for OE.

**Conclusions:**

Using chitosan nanoparticles encapsulated with EO presents an effective treatment approach for dogs with OE that conventional antimicrobial treatments have not cured. This approach not only enhances antibacterial effects but also reduces the required dosage of antimicrobials, potentially preventing the emergence of antimicrobial resistance.

## Background

Ajwain, scientifically known as *Trachyspermum ammi* (*T. ammi*), belongs to the *Apiaceae* family and the *Apiales* order. The well-known *T. ammi* fruit or dried seeds are regarded as a highly nutrient-dense or therapeutically enhanced component of the plant [1–3]. In medieval Persian medicine and pharmaceutical manuscripts, Ajwain is called Zenyan or Nankhah. It has been utilized in Persian medicine for thousands of years. The plant contains several bioactive compounds of pharmacological significance, including saponins, phenolic compounds, carbohydrates, fats, fiber, volatile oil, glycosides, and minerals. In modern medicine, Ajwain has been used for various purposes such as bronchodilation, cardiac stimulation, carminative effects, digestive stimulation, diuretic properties, Galactagogue, hypoglycemic effects, anti-inflammatory properties, analgesic effects, antibiotic activity, and as an ant filarial agent [1]. The essential oil (EO) extracted from the dried seeds of *T. ammi* contains approximately 50% Thymol, a potent germicide, anti-spasmodic, and fungicide. Thymol is considered a fourth-generation herbal antibiotic as it can effectively kill bacteria resistant to common third-generation antibiotics and multi-drug-resistant microbial pathogens. Ajwain essential oil also contains phenol, benzene methyl, and γ-terpinene, which exhibit antibacterial activity similar to Thymol [4].

Numerous studies have extensively investigated the antimicrobial properties of EOs and their constituents. However, EOs are volatile and delicate compounds prone to enzymatic reactions and susceptible to degradation when exposed to oxygen, light, moisture, and heat. These factors can compromise their biological properties, leading to decreased activity and increased toxicity, which limits their traditional use [5, 6]. To overcome the limitations and challenges associated with EOs, researchers have explored the encapsulation of EOs within drug delivery systems. This approach allows for the controlled release of the active compounds, enhancing the bioavailability and efficacy of the EOs and achieving an optimal pharmacokinetic profile [5, 7].

Chitosan (CS) and its derivatives have found remarkable applications in the biomedical field, particularly in the controlled release of active substances. CS is commonly modified to form a hydrogel (HG) for this purpose [8, 9]. Nano-gels, on the other hand, are HGs that possess nanoscale dimensions. A HG is a cross-linked polymer chain network forming a macromolecular structure [10]. These cross-linked polymeric networks, known as HG, can mimic soft tissues and create a moist environment. CS, a linear polysaccharide, demonstrates pH-responsive self-assembling properties. It is preferred for constructing HGs due to its inherent intermolecular and intramolecular hydrogen bonding capabilities and its versatile reactive amino groups [11].

Canine Otitis Externa (OE) is a common skin disease affecting dogs, accounting for approximately 20% of small-animal counseling cases [12–14]. This condition is multifactorial, and it manifests with various clinical signs, including head shaking, ear scratching, ceruminous or purulent discharge, self-inflicted skin lesions, malodor, swelling, and pain [15]. *Staphylococcus* spp. is the most frequently isolated bacterial species from the ear canals of dogs with OE. However, other bacteria, such as *Streptococcus* spp., *Pseudomonas* spp., *Corynebacterium* spp., *Escherichia coli*, and other *Enterobacteriaceae*, can also be involved [16, 17]. Diagnosis of OE typically involves palpation of the ear canal, visual inspection, and Otoscope examination. Cytology and bacterial culture are commonly used diagnostic tools. In cases of colonization by opportunistic bacteria, veterinarians may face therapeutic challenges [16, 18].

Topical antimicrobial therapy is the preferred treatment approach for OE, as systemic medications are less effective. However, conventional drugs often encounter bacterial resistance. Hence, EOs are being explored as alternative therapies, and further studies are needed to determine their efficacy against the bacteria that cause OE [15, 17]. In this study, Nano-gels were produced by creating amid linkages between the amino groups in CS and the carboxyl group of Cholesterol (CHOL). The study aimed to investigate the potential effects of *T. ammi* EO encapsulated in CS-CHOL Nano-gels as a green carrier against bacterial pathogens isolated from cases of OE. The current study seeks to provide a solution to the pressing demand for therapeutic alternatives in treating infections caused by antibiotic-resistant bacteria.

## Results

### Direct cytology results

Based on the results of cytological analysis of swabs obtained from dogs suffering from OE, the frequency of gram-positive cocci, gram-negative rods, gram-positive rods, and gram-positive cocci/rods were 58.66%, 33.33%, 1.33%, and 6.66%, respectively.

## Bacterial culture results

A total of 75 bacterial species were isolated from dogs with OE, indicating their involvement in causing the condition. Among these, *Staphylococcus pseudintermedius* was the most frequently observed bacterium, with a frequency of 37 (49.3%). Following *S. pseudintermedius*, the next most commonly identified bacteria were *Pseudomonas aeruginosa* (11 (14.7%)), *Escherichia coli* (10 (13.3%)), *Streptococcus canis* (7 (9.3*%)), Corynebacterium auriscanis* (5 (6.7%)), *Klebsiella pneumoniae* (2 (2.7%)), *Proteus Mirabilis* (2 (2.7%)), and *Bacillus cereus* (1 (1.3%)). The isolated bacterial species were identified based on several criteria, including Gram morphology, culture morphology, and specific biochemical tests associated with each species. The identification process followed the guidelines and references provided by Markey et al. and Quinn, widely recognized references in bacterial identification [19, 20].

### Antibiotic susceptibility results

No antibiotic can provide 100% effectiveness against all isolated bacteria. However, the isolated bacteria in this study demonstrated satisfactory susceptibility to certain antibiotics. Specifically, they showed sensitivity to Amikacin, Gentamicin, Cephalothin, and Ceftriaxone. *S. pseudintermedius* exhibited high sensitivity to Amikacin, Cephalothin, and Gentamicin while showing notable resistance to Penicillin G, Ampicillin, and Oxytetracycline. In general, Penicillin G, Erythromycin, and Ampicillin demonstrated the highest levels of antibiotic resistance among the bacteria examined (Table 1). The quality control strains used in the study yielded results following the guidelines set by the CLSI, indicating that the test conditions were appropriate and reliable [21].

**Table 1:** Antimicrobial resistance percentage of isolated bacteria from dogs with OE

Bacteria	Percentage of Resistance for Each Antibiotic									
	P5	Enr5	Kf30	Amp2	Ot30	Rd5	E15	Ak30	Cn10	Cro30
*S. pseudintermedius*	64.86	40.54	2.7	56.8	37.8	21.6	29.7	0	8.1	5.4
*E. coli*	80	70	40	80	30	50	20	10	10	20
*P. aeruginosa*	54.5	81.8	36.4	63.6	72.7	45.4	9.1	9.1	27.3	18.2
*P. Mirabilis*	100	100	50	50	50	50	50	0	50	50
*S. canis*	28.6	42.9	0	14.3	57.1	14.3	28.6	0	14.3	28.6
*C. auriscanis*	100	100	40	100	40	60	40	0	0	0
*K. pneumoniae*	100	50	0	0	0	100	50	0	0	0
*B. cereus*	100	100	0	100	0	0	100	0	0	0

P5, Penicillin G; E15, Erythromycin; Kf30, Cephalothin; Amp2, Ampicillin; Ot30, Oxytetracycline; Rd5, Rifampcin; Enr5, Enrofloxacin; Ak30, Amikacin; Cn10, Gentamicin; Cro30, Ceftriaxone

### GC/MS analysis results

Using gas chromatography/mass spectrometry (GC/MS) analysis, the study identified 11 compounds present in *T. amm*i EO, which was extracted in the present study (Table 2). Among these compounds, Thymol was present in a notably high percentage, accounting for 48% of the EO composition.

**Table 2:** Chemical analysis of EO of *T. ammi* by GC/MS

SN.	Components	Percentage	Formula	`RT*
1.	Thymol	48	C_10_H_14_O	78.23
2.	*p*-Cymene	23	C_10_H_14_	45.17
3.	γ-Terpinene	17	C_10_H_16_	43.43
4.	*β*-Pinene	1.3	C_10_H_16_	17.26
5.	*β* -Phellandrene	0.6	C_10_H_16_	13.26
6.	Sabinene	0.8	C_10_H_16_	12.19
7.	*β*-Myrcene	0.5	C_10_H_16_	10.38
8.	*α*-Terpinene	0.4	C_10_H_16_	11.62
9.	Terpinene	0.4	C_10_H_16_	10.23
10.	*α*-Pinene	0.3	C_10_H_16_	7.84
11.	*α*-Thujene	0.2	C_10_H_16_	6.78

### SEM & successful encapsulating of EO in CS-CHOL nano-gel

**Fig. 1:**
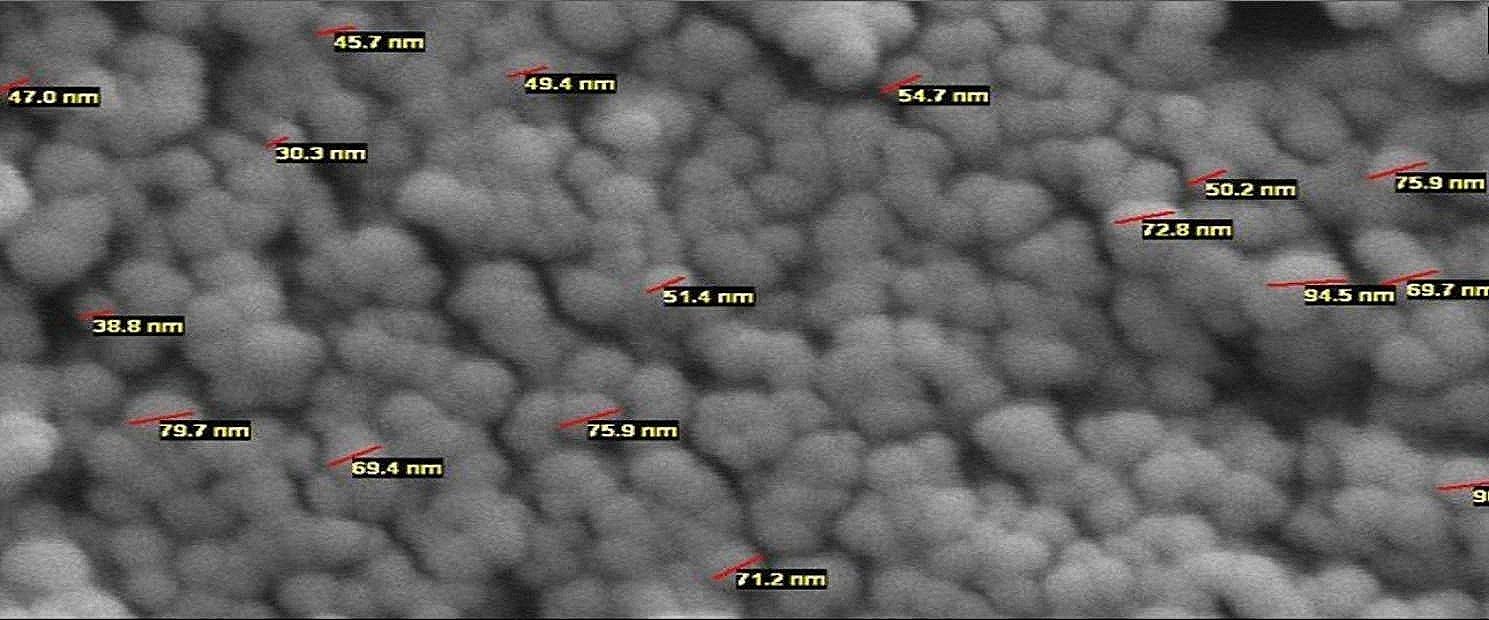
Scanning electron microscopy (SEM) image of CS-CHOL Nano-gel

SEM analysis revealed that the produced Nano-gels exhibited a diameter of less than 100 nm, indicating their nanoscale size. Furthermore, the Nano-gels displayed a uniform and spherical morphology (Fig. 1). The encapsulation efficiency of the EO was determined to be 67.23%, indicating a substantial accumulation of the EO within the Nano-gels.

### Antibacterial Effect of free and encapsulated EO based on disk diffusion method

**Fig. 2:**
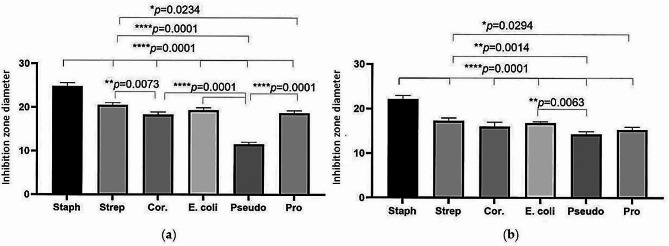
The diagram of the inhibition zone diameter of the selected microbial strains against the disc containing *T. ammi* EO encapsulated in CS-CHOL Nano-gel (**a**) and a disc containing *T. ammi* EO (**b**). P, P-value; Staph, *S. pseudintermedius*; Strep, *S. canis*, Cor, *C. auriscanis*; Pseudo, *P. aeruginosa*; Pro, *P. Mirabilis*

The findings indicate that the antibacterial effects of *T. ammi* EO encapsulated in CS-CHOL Nano-gel are more pronounced than those of the free form of *T. ammi* EO (Fig. 2). Negative control discs containing CS-CHOL Nano-gel alone did not affect the six bacterial strains tested. The most significant inhibitory effect of *T. ammi* EO was observed against *S. pseudintermedius*. On the other hand, the lowest inhibitory effect of *T. ammi* EO encapsulated in CS-CHOL Nano-gel was observed against *P. aeruginosa* (Fig. 2). The results were considered significant when the p-value was less than 0.05 for the *T. ammi* EO and *T. ammi* EO encapsulated in CS-CHOL Nano-gel groups.

### MIC and MBC findings

**Fig. 3:**
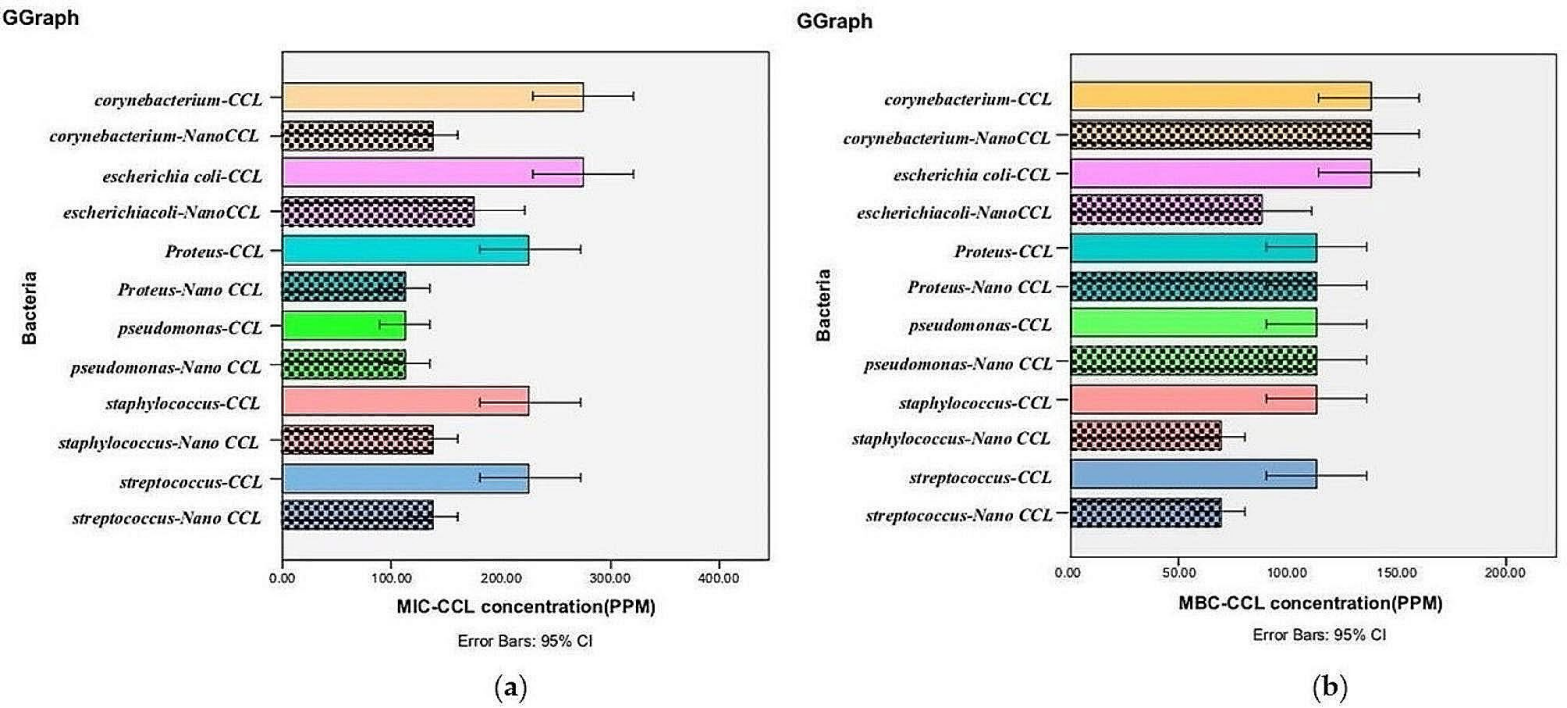
The MIC of *T. ammi* EO encapsulated in CS-CHOL Nano-gel (Name of bacteria-Nano CCL) and CS-CHOL Nano-gel without *T. ammi* EO (Name of bacteria-CCL) on the selected bacteria (**a**). The MBC of *T. ammi* EO encapsulated in CS-CHOL Nano-gel (Name of bacteria-Nano CCL) and CS-CHOL Nano-gel without *T. ammi* EO (Name of bacteria-CCL) on the selected bacteria (**b**)

The results of MIC tests showed a more significant inhibitory effect of *T. ammi* EO encapsulated in CS-CHOL Nano-gel compared to CS-CHOL Nano-gel without *T. ammi* EO (*P* < 0.05) (Fig. 3-a) and *T. ammi* EO (*P* < 0.05) (Table 3). In both repetitions of the experiment, the results were the same. By comparing the MIC of *T. ammi* EO and CS-CHOL Nano-gel with *T. ammi* EO, with *S. pseudintermedius*, *S. canis*, *C. auriscanis*, *E. coli*, and *P. Mirabilis*, the P-value was less than 0.05, and significant results were reported. Comparison MBC of *T. ammi* EO and *T. ammi* EO encapsulated in CS-CHOL Nano-gel for *S. pseudintermedius*, *S. canis*, and *E. coli* bacteria reported significant results (Table 3).

*T. ammi* EO encapsulated in CS-CHOL Nano-gel had an excellent inhibitory effect on *S. pseudintermedius*, *S. canis*, *P. aeruginosa*, and *P. Mirabilis*, so the mean MIC for these bacteria was 112.5 ppm. The mean MIC for *C. auriscanis* and *E. coli* was 137.5 ppm, which indicates that higher amounts of *T. ammi* EO encapsulated in CS-CHOL Nano-gel are needed. Among the tested bacterial samples, *S. pseudintermedius* and *S. canis* were the most sensitive to *T. ammi* EO encapsulated in CS-CHOL Nano-gel (Table 3), and their mean MIC was 68.75 ppm. CS-CHOL Nano-gel with *T. ammi* EO shows a satisfactory bactericidal effect (MBC) for *S. pseudintermedius* and *S. canis* (Fig. 3-b). In general, there is little difference between MIC and MBC values.

**Table 3:** Statistical analysis and comparison of MIC and MBC of *T. ammi* EO and *T. ammi* EO encapsulated in CS-CHOL Nano-gel with each selected bacteria separately by T-test

Indicator		S. pseudintermedius	S. canis	C. auriscanis	E. coli	P. Mirabilis	P. aeruginosa
MIC	EO^*^	0.002	0.002	0.001	0.003	0.001	1
	CS-EO^**^	0.004	0.004	0.001	0.003	0.002	1
MBC	EO	0.002	0.002	1	0.003	1	1
	CS-EO	0.004	0.004	1	0.003	1	1

^*^EO, *T. ammi* EO; ^**^CS- EO, *T. ammi* EO encapsulated in CS-CHOL Nano-gel

## Discussion

Various studies have employed different cross-linking methods to synthesize Nano-gels to microencapsulate EOs. These methods include self-assembly, ionic cross-linking, cross-linking polymerization, crystallization, radiation cross-linking, and functional group cross-linking. Each method offers unique advantages and can be tailored to meet specific requirements regarding the desired properties and applications of the Nano-gels [22]. The present study utilized self-assembly to create Nano-gels composed of CS polymer modified with cholesterol. These Nano-gels possess unique characteristics such as high biocompatibility, low toxicity, and biodegradability, making them suitable for producing nanometer-sized carriers for microencapsulating EOs [8]. CS is a polymer derived from acetyl glucosamine and is obtained by partially deacetylating chitin through alkaline treatment [23]. CS has limited surface activity due to the absence of hydrophobic segments. However, the surface activity of CS can be enhanced through chemical modifications [24].

The hydrophilic nature of chitosan (CS) is a crucial characteristic that allows for creation of self-assembled nanoparticles by incorporating hydrophobic components into CS. This property makes CS well-suited for applications in drug delivery. The hydrophobic cavities within CS can serve as storage compartments for various bioactive materials. The drug’s therapeutic efficiency can be improved by binding targeted components to the drug-loaded nanoparticles’ surface [8, 25]. In synthesis, non-polar cholesterol is bound to the CS polymer using EDC (Cross-linker). When this complex is placed in a polar environment, the non-polar heads of cholesterol accumulate inside the complex. In contrast, the polar heads are oriented towards the outside, forming a spherical complex structure. This connection has been confirmed through electron microscopy [26].

The present study confirmed the shape and size of the Nano-gels through SEM imaging, which revealed well-separated spherical nanoparticles without any aggregation. This lack of aggregation can be attributed to the positive charges on the CS, which prevent the Nano-gels from sticking together. Previous research by Kosaraju et al. (2006) has reported that interactions between polyphenols and matrix polymers can result in smooth surface particles. Therefore, intermolecular or intramolecular interactions may contribute to the relatively smooth surface of the nanoparticles [27].

The antimicrobial properties of *T. ammi* EO have been demonstrated in various studies [28]. The present study analysis of the EO revealed that the main components were Thymol (48%) and *p*-Cymene (23%). These compounds contribute significantly to the antimicrobial activity of *T. ammi* EO, which is consistent with findings from other research studies. In 2023, Mishra et al. identified Thymol as the primary component of *T. ammi* EO through GC-MS analysis [29]. Researchers have also investigated the antibacterial mechanism of Thymol. It has been found that Thymol increases the permeability of the bacterial cell membrane. It can disrupt the outer membrane of bacteria, leading to the release of lipopolysaccharide (LPS) and increased cytoplasmic permeability to ATP [30, 31].

In the current study, the loading capacity of *T. ammi* EO in the Nano-gel was determined to be 67.23%, indicating successful storage of the EO in the Nano-gel. The structure of the Nano-gel was also found to protect the EO and control its release effectively. The durability of EO-containing nanoparticles can be attributed to the slow and stable release of active components within the nanoparticles [32]. Previous research by Yang et al. in 2009 demonstrated that nanoparticles containing garlic EO remained stable for up to five months [33]. Recent studies have highlighted the substantial potential of nanoparticles functionalized with EOs for antimicrobial activity against multidrug-resistant pathogens. This antimicrobial activity is attributed to the improved stability and solubility of EOs and the reduced evaporation and degradation of active EO components [6]. In 2022, Zarenejad et al. combined *T. ammi* EO with CS nanoparticles and compared it with free EO, showing better efficiency of the EOs combined with nanoparticles [34].

The free and encapsulated *T. ammi* EO demonstrated excellent inhibitory activity against *S. pseudintermedius* and *S. canis*. The growth-inhibiting activity of the encapsulated *T. ammi* EO was more pronounced than that of the free EO against *P. Mirabilis* and *C. auriscanis*. Statistical analysis of the MIC and MBC results in the present study indicated that the encapsulated *T. ammi* EO exhibited a strong antibacterial effect against Gram-positive bacteria, such as *S. pseudintermedius* and *S. canis*, as well as Gram-negative *E. coli*. Furthermore, no significant difference was observed in the MIC and MBC results between the CS-CHOL Nano-gel without *T. ammi* EO, and *T. ammi* EO encapsulated in CS-CHOL Nano-gel in *P. Mirabilis* species. Similar findings were reported by Zarenejad et al. in 2022, where no substantial difference in effectiveness was observed in *Pseudomonas* spp. when evaluating the efficacy of EOs. The reduced antibacterial effect of *T. ammi* EO against Gram-negative bacteria can be attributed to the unique cell walls of these bacteria, which provide them with greater resistance to the antimicrobial activity of the EO [34].

Several studies conducted on OE have reported findings similar to the current research regarding the isolation of infectious agents and their antibiotic resistance patterns. In 2023, Abani et al. isolated *Staphylococcus* spp., *Streptococcus* spp., *Pseudomonas* spp., and *Enterobacteriaceae* spp. from cases of OE in dogs, and these isolates showed evident antibiotic resistance, similar to the findings of the present study [17]. Similarly, in 2016, De Martino et al. updated the microbiological causes of Otitis in dogs in the Campania region of Italy. They identified *Staphylococcus* spp., *Pseudomonas* spp., *E. coli*, and *Proteus* spp. as important pathogens causing ear infections in dogs. In this study, the isolated bacterial strains demonstrated a significant level of resistance to at least four classes of antimicrobial drugs that were utilized [35]. Bugden conducted a study in 2012 on bacteria isolated from the ears of 3,541 dogs with Otitis in Australia. The most commonly isolated bacteria were *Pseudomonas* spp. (35.5%), *Staphylococcus* spp. (24.3%), *Proteus* spp. (6.8%), *streptococci* spp. (6.2%), and *E. coli* (4.2%). Most isolates showed sensitivity to Gentamicin but were highly resistant to Polymyxin B treatment. *Streptococci* spp. also exhibited increased resistance to all antibiotics used in the study [36]. In the present study, among the total of 75 isolated bacteria, *S. pseudintermedius* accounted for 49.3%, *P. aeruginosa* for 14.7%, *E. coli* for 13.3%, *S. canis* for 9.3%, and *C. auriscanis* for 7% of the total isolates. These findings are consistent with the prevalence of these bacterial species in cases of OE [17, 35, 36].

In order to mitigate the adverse effects and potentially reverse antimicrobial resistance, one effective treatment strategy is to reduce the usage of antimicrobials. Additionally, the development of drug combinations has emerged as a novel approach to controlling resistant pathogens. Combining antimicrobials with EOs against resistant bacteria can broaden the antimicrobial spectrum, thereby reducing the emergence of resistant variants and minimizing the reliance on a single antimicrobial agent [37, 38]. For instance, in a study conducted by Rosato et al. in 2007, the use of *Pelargonium graveolens* EO was found to reduce the minimum effective dose of Norfloxacin against *B. cereus*, *B. subtilis*, *E. coli*, and *Staphylococcus* spp., demonstrating an apparent synergistic effect [39]. Other studies have reported that EOs such as *Eremanthus erythropappus* and *Origanum vulgare* can enhance the action of beta-lactam antibiotics against both Gram-positive and Gram-negative bacteria that produce beta-lactamase enzymes [37].

## Conclusion

There is an undeniable and urgent need for alternative treatment options for infections caused by antibiotic-resistant bacteria. Addressing this issue requires proactive initiatives and efforts from the scientific and medical communities. Developing novel therapies, exploring alternative antimicrobial agents, promoting antimicrobial stewardship programs, and implementing infection prevention and control measures are some of the key strategies that can help combat the challenge of antibiotic resistance. It is crucial to prioritize research and funding in this area to ensure the availability of effective treatment options for patients affected by antibiotic-resistant infections. The present study’s findings support the effectiveness of *T. ammi* EO in HGs against the pathogens responsible for OE. The superior antibacterial effects observed with EO encapsulated in HGs, compared to free EOs and CS-CHOL Nano-gel, indicate the potential of such HGs in combination with other commonly used antimicrobials for treating OE. This approach offers a way to reintroduce EOs and antimicrobials that may have been abandoned due to widespread resistance. Combining these agents in HGs can enhance the antibacterial effects, and the required dosage of antimicrobials may be reduced, potentially preventing the emergence of antimicrobial resistance.

## Methods

### Materials

Polysaccharide Chitosan (Sigma, USA) with low molecular weight and about 8% degree of deacetylation was purchased; Brain heart infusion (BHI), mannitol salt agar (MSA) and Mc-Conkey (Merck, Germany); Mueller-Hinton agar (MHA) (Oxoid, UK); API-Coryne system (BioMerieux, France); 1-Ethyl-3-(3-dimethylaminopropyl)carbodiimide (EDC), Cholesterol, Acetic acid, Dimethyl sulfoxide (DMSO), blank paper discs were obtained from Padtan-Teb Company, Iran; Commercial antibiotic disks (Oxoid Ltd, UK); and fruit of *T. ammi* plant was purchased from Pakan Seed Company (Isfahan, Iran) with herbarium stock number, 293-0303-1.

### Animals

Investigations were conducted into fifty-seven dogs with OE referred to the Veterinary Hospital of Small Animals. The most important signs in the history of affected animals included itching of the ear, abnormal secretions, and malodorous ears. In clinical examination, symptoms such as redness of the auricle and external ear canal, pain in palpation of the ear, abnormal secretion, and malodorous ear were observed. OE was confirmed in all cases by otoscope and cytological examination. Animals with any history of other diseases or antibiotic therapy within the last two months were excluded from this study. If several cases from the same housing were referred with OE, sampling was conducted from only one.

### Sampling & bacteriological process

Samples were collected from dogs that exhibited signs of OE, including ear itching, abnormal secretions, and odor. The sampling procedure involved using sterile swabs to collect samples from the end of the vertical part of the external ear. Three sterile swabs were taken to prevent contamination before conducting an otoscope examination. Each swab was used for different analyses, including cytological and bacterial cultures. A third swab was replaced if any swabs became contaminated or damaged during the procedure. Care was taken to ensure that the swab made minimal contact with the outer portion of the ear canal and the surrounding hairs when entering and exiting during the sampling process. The swab was rotated 360° inside the ear canal for approximately five seconds. Following sample collection, the swabs were rolled onto cytological slides and fixed with heat. Gram staining was performed on the slides and was subsequently evaluated microscopically. The swabs were sent to a laboratory within two hours for further analysis.

The process related to bacteriology, including aerobic cultivation in blood agar (Columbia agar with 5% sheep blood), BHI, MSA, and MacConkey. The cultures were incubated at 37 °C for 24 to 48 h. Bacterial species were identified by Gram morphological and biochemical characteristics (TSI, Urease test, IMViC, Motility test, Lecithinase Test, oxidase, Catalase, Nitrate reduction, and Coagulase.), as described by Murray et al. and Markey et al. [20, 40]. . *Corynebacterium* spp. was identified by the API-Coryne system according to the manufacturer’s instructions.

### Preparation of *T. ammi* EO

The fruit of the *T. ammi* plant was purchased, and after confirming the purity of the seed, the EO was extracted from the milled powder of the fruit by steam distillation using a Clevenger machine. EO was dehydrated with sodium sulfate. It was kept in a dark glass container with a closed lid, away from light, and at a cool temperature [41].

### GC/MS analysis of EO

The EO compounds were identified using GC/MS. The system consisted of an Agilent 6890 gas chromatograph with an Agilent 5973 mass-selective detector (Agilent, USA). Since the compounds in EOs are known as volatile substances in terms of molecular weight and polarity, the process of separating and identifying the components of EO was carried out by gas chromatography combined with mass spectrometry.

### Synthesis of chitosan nano-gel

The Nano-gel was prepared using a self-assembly method involving CS and cholesterol (CHOL) with the assistance of an EDC cross-linker [42, 43]. To prepare a 0.5% CS solution, 1 gr of CS was added to 200 µl of a 1% acetic acid solution (v/v). The mixture was then placed on a magnetic stirrer and stirred at a speed of 500 rpm for 20 min. The resulting solution, which had a semi-transparent appearance and a fragile yellow color, was subjected to homogenization using an ultrasonic sonicator (Elmasonic, E30H, Germany) for 20 min. The sonicator was operated at a power of 70 watts. After the homogenization process, a stock solution with a concentration of 5000 ppm was obtained.

To incorporate CHOL into the CS solution, 215 mg of CHOL was initially dissolved in 10 µl of ethanol. Subsequently, 178 µl of EDC was added to the CHOL solution, and the mixture was kept in darkness for a short period. To the formation of amid linkages between the carboxyl groups of CHOL (C_26_H_45_COOH) and the amino groups of CS (*a* [1–4]-2-amino-2-deoxy *B*-D-glucan), the CHOL-EDC mixture was added dropwise, slowly, to a solution containing 75 µl of CS while stirring on a magnetic stirrer. To ensure a complete formation of amid linkages between CS and CHOL, The resulting mixture was left to stir on a magnetic stirrer for 24 h to allow for adequate linkage formation. Next, the pH of the mixture was adjusted to a range of 8.5-9 by adding sodium hydroxide while continuing to mix on a magnetic stirrer. As the pH gradually increased, gel formation was observed. To separate the formed Nano-gel particles, the mixture was centrifuged for 5 min at a speed of 9000 rpm. This centrifugation step aimed to separate the Nano-gel particles from the rest of the solution. Following the centrifugation step, the supernatant solution was carefully separated from the white Nano-gel deposits that had formed on the wall of the centrifuge tube. The centrifugation process was repeated twice using ethanol to eliminate impurities that did not participate in the reaction, including EDC, CS, and CHOL. After each centrifugation, the supernatant solution was discarded.

The resulting Nano-gel particles were dispersed in a 1% acetic acid solution. The dispersion was filtered using a filter with a pore size of 0.2 mm to obtain a uniform particle size. This filtration step helped to remove any larger particles or aggregates from the Nano-gel suspension. Subsequently, the filtered Nano-gel particles were dried thoroughly using a vacuum dryer. The dried Nano-gel was then stored in a dark glass container at a cool temperature.

SEM was employed to investigate the morphology and size of the CS-CHOL Nano-gel. The SEM analysis was conducted at an accelerating voltage of 70 kV to obtain detailed information about the structure and size distribution of the Nano-gel particles.

### Encapsulation of *T. ammi* EO in CS-CHOL nano-gel

A total of 0.5 µl of *T. ammi* EO was dissolved in 1 µl of DMSO, then 0.5 µl of the solution was diluted with deionized water to reach a final volume of 10 µl. This solution was named Solution Number 1. CS-CHOL Nano-gel (1 mg) was added to 1 µl of Solution Number 1 and kept for 24 h at room temperature. It was centrifuged for 15 min at 12,000 rpm and 20 °C to separate the particles. In the end, two separate phases were created; the supernatant was discarded, and the sediment containing CS-CHOL Nano-gel encapsulated with *T. ammi* EO was used for microbial tests. The optical absorbance of the supernatant was taken by UV-Vis spectrophotometry using а Varioskan Flash Multimode Reader (Thermo Scientific, Waltham, MA) at a wavelength of 280 nm to calculate the Loading efficiency of the EO according to the formula [1].

1 $$Loading\;Efficiency \% =\left[\frac{OD r-OD s }{OD r}\right]\times 100$$

### Chosen isolates of bacteria

Six bacterial strains that exhibited higher abundance in dogs affected by OE were selected for the next steps: *S. pseudintermedius*, *S. canis*, *P. aeruginosa*, *E. coli*, *C. auriscanis*, and *P. Mirabilis*. These strains were preserved in glycerol broth and stored at -80 °C. The selected strains were utilized to examine the impact of free EO and encapsulated EO.

### Antimicrobial susceptibility tests

**Fig. 4:**
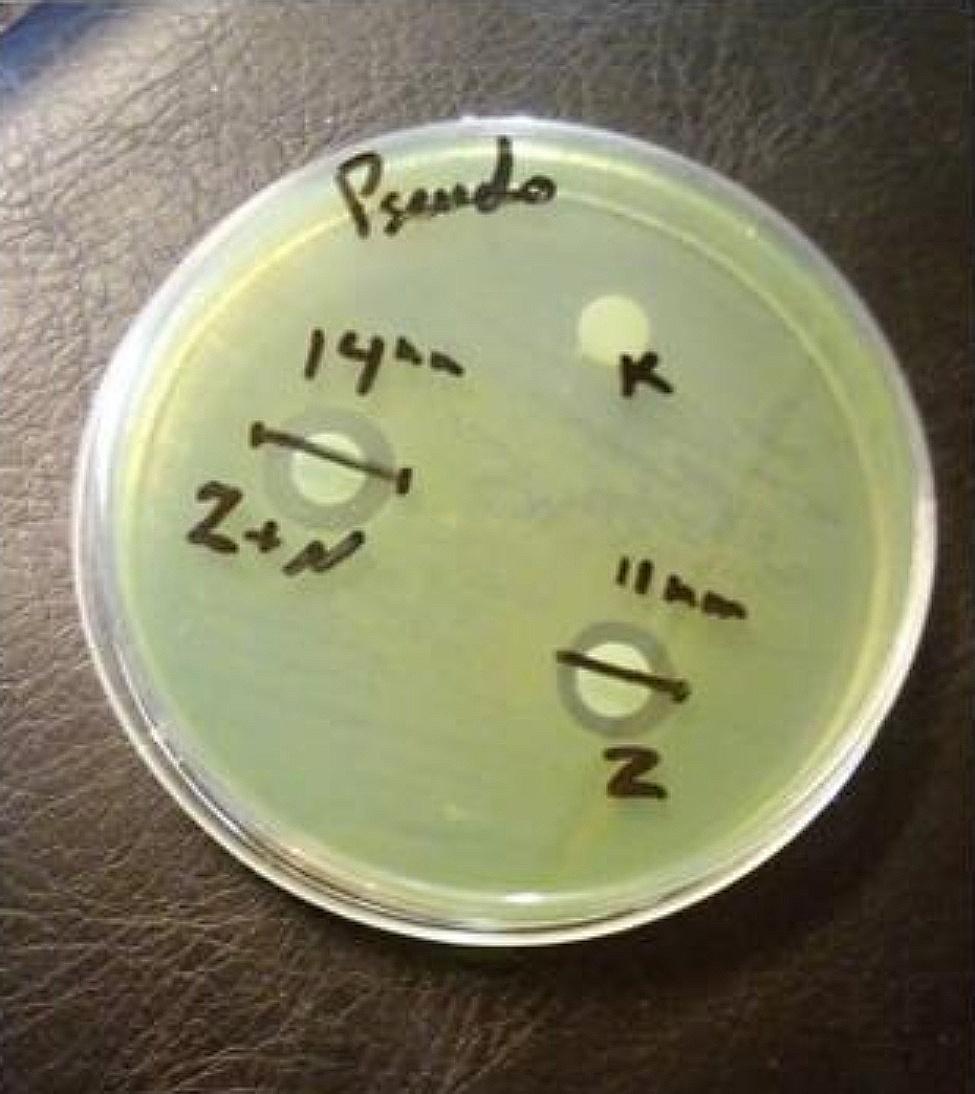
Determination of the antimicrobial sensitivity of the EO by the disc diffusion method on *Pseudomonas*. **K**: CS-CHOL Nano-gel as a negative control disk; **Z**: free *T. ammi* EO; **Z + N**: *T. ammi* EO in CS-CHOL Nano-gel

In order to assess the antibacterial activity, 30 µl of *T. ammi* EO and *T. ammi* EO in CS-CHOL Nano-gel (100 µg ml^− 1^) were separately inoculated onto blank paper discs. These discs serve as a means to release antibacterial substances into the culture medium containing the targeted bacteria. Subsequently, MHA plates were prepared under standard conditions and inoculated with microbial strains isolated from cases of OE using surface culture plating. The paper discs were then placed onto the agar plates and incubated at 37 °C for 18–24 h. After incubation, the antibacterial susceptibility was evaluated by measuring the diameter of the inhibition zones around the discs. The negative control was a blank disc coated with unencapsulated CS-CHOL Nano-gel (Fig. 4).

The antimicrobial susceptibility patterns of the isolated bacterial strains were determined using the disk diffusion test on MHA. The test was performed following the recommendations of the Clinical Laboratory Standards Institute (CLSI) as described in the 33rd Edition of the M100 guidelines (2023) [21]. Commercial antibiotic disks (Oxoid Ltd, UK) containing the following drugs and concentrations were used: Amikacin (Ak30; 30 µg), Ampicillin (Amp2; 2 µg), Ceftriaxone (Cro30; 30 µg), Cephalothin (Kf30; 30 µg), Gentamicin (Cn10; 30 µg), Enrofloxacin (Enr5; 5 µg), Erythromycin (E15; 15 µg), Oxytetracycline (Ot30; 30 µg), Rifampicin (Rd5; 5 µg), and Penicillin G (P5; 5 units). The selection of drugs was based on the strains and their clinical use.

After incubation for 24 h at 37 °C, the diameters of the inhibitory zones around the antibiotic disks were measured and evaluated according to CLSI guidelines. The strains were classified as sensitive or resistant to the drug, with intermediate susceptibility considered resistant. Only the percentage of resistance is reported in the results. Strains obtained from the American Type Culture Collection (ATCC), such as E. coli 25,922, S. aureus 25,923, S. pneumoniae 49,619, and P. aeruginosa 27,853, were used for quality control.

### Determination of MIC and MBC values

The MIC of encapsulated *T. ammi* EO in CS-CHOL Nano-gel against the bacteria isolated from cases of OE in dogs was determined using the in vitro broth microdilution method. The experimental protocol used in the present study was based on the methods described by Sharifi and Nayeri Fasaei [41] and Firmino et al. [44]. The MIC was determined in 96-well polystyrene plates. For this test, 100 µg of *T. ammi* EO in CS-CHOL Nano-gel solution under aseptic conditions was serially diluted in a BHI to attain final concentrations of 25 to 0.19 µg ml^− 1^. Next, 100 µl of bacterial culture previously set at a concentration of ∼ 5 × 10^5^ CFU ml^− 1^ was added.

Each bacterial isolate was subjected to individual testing in a separate experiment, and each experiment was repeated twice. Two wells were utilized in each microplate for the positive and negative controls. The positive control consisted of 100 µl of BHI broth and 100 µl of microbial suspension. Conversely, the negative control contained 100 µl of BHI broth and 100 µl *T. ammi* EO in CS-CHOL Nano-gel. The microplates were then incubated aerobically at 37 °C for 24 h. After incubation, the wells were visually examined, and the optical absorbance was measured using an ELISA reader. The MIC was determined to be the lowest concentration of *T. ammi* EO in CS-CHOL Nano-gel, at which no visible growth was detected in the wells. To assess the effects of the CS-CHOL Nano-gel without *T. ammi* EO, the MIC was determined individually for each bacterium.

The MIC of the EO alone was also determined to compare the effects of *T. ammi* EO alone with the effects of the EO encapsulated in CS-CHOL Nano-gel. To enhance the solubility of *T. ammi* EO, it was diluted 1:10 with a 1.5% DMSO solution. DMSO is widely recognized as non-toxic when used at concentrations below 10% (v/v) [45]. To determine the MBC, 50 µl of each well that exhibited no visible growth in the MIC test were cultured on a blood agar medium and incubated at 37 °C for 24 h. The MBC was defined as the lowest concentration at which the original bacterial growth was reduced by a minimum of 99.9% [44, 46].

### Statistical analysis

The collected data was analyzed using SPSS 25.0 software (SPSS Inc., Chicago, IL). A comparison between *T. ammi* EO, CS-CHOL Nano-gel encapsulated with *T. ammi* EO, and CS-CHOL Nano-gel without *T. ammi* EO was conducted using an independent t-test with four repetitions.

## Data Availability

All relevant data is contained within the manuscript. The data generated and analyzed during the current study are available from the corresponding author on request.

## Abbreviations

*T. ammi*: *Trachyspermum ammi*
*EO*: Essential Oil
HG: Hydrogel
CS: Chitosan
OE: Otitis Externa [inflammation of the external ear canal
DMSO: Dimethyl sulfoxide
CHOL: Cholesterol
EDC: Ethylcarbodiimide hydrochloride
SEM: Scanning Electron Microscopy
GC/MS: Gas Chromatography-Mass Spectrometry
